# A Deep Learning Framework for Segmenting Brain Tumors Using MRI and Synthetically Generated CT Images

**DOI:** 10.3390/s22020523

**Published:** 2022-01-11

**Authors:** Kh Tohidul Islam, Sudanthi Wijewickrema, Stephen O’Leary

**Affiliations:** Department of Surgery (Otolaryngology), Faculty of Medicine, Dentistry and Health Sciences, University of Melbourne, Melbourne, VIC 3010, Australia; swijewickrem@unimelb.edu.au (S.W.); sjoleary@unimelb.edu.au (S.O.)

**Keywords:** deep learning, medical image processing, image fusion, segmentation, brain tumor

## Abstract

Multi-modal three-dimensional (3-D) image segmentation is used in many medical applications, such as disease diagnosis, treatment planning, and image-guided surgery. Although multi-modal images provide information that no single image modality alone can provide, integrating such information to be used in segmentation is a challenging task. Numerous methods have been introduced to solve the problem of multi-modal medical image segmentation in recent years. In this paper, we propose a solution for the task of brain tumor segmentation. To this end, we first introduce a method of enhancing an existing magnetic resonance imaging (MRI) dataset by generating synthetic computed tomography (CT) images. Then, we discuss a process of systematic optimization of a convolutional neural network (CNN) architecture that uses this enhanced dataset, in order to customize it for our task. Using publicly available datasets, we show that the proposed method outperforms similar existing methods.

## 1. Introduction

Although vast amounts of medical images are produced each day, most are still interpreted through visual analysis on a slice-by-slice basis [1]. This requires experience, is time consuming, expensive, prone to human error, and most importantly, is inadequate for the processing of large-scale specimens [2]. Automated image processing/analysis techniques have gained popularity as alternatives that overcome these concerns.

Segmentation is a standard method of image analysis where an image is separated into different regions, typically based on the characteristics of its pixels (for two-dimensional (2-D) images) or voxels (for three-dimensional (3-D) volumes) [3]. Applications of segmentation in medical imaging include the identification of pixels/voxels representing organs [4] and pathologies such as tumors [5]. Here, we focus on the latter application: the segmentation of tumors. Using information from multiple imaging modalities can enhance the segmentation process, as different modalities provide different information on target regions [6]. As such, multi-modal medical image segmentation has the potential to deliver more reliable and accurate segmentation results. However, integrating such information efficiently still remains a challenge [7].

Deep learning techniques have gained considerable interest in the field of multi-modal medical image segmentation due to their self-learning and generalization ability over large-scale data [8]. Furthermore, such methods have shown improved performance when compared to single-mode segmentation methods in many instances [9]. As multi-modal images originate from different imaging sources, some pre-processing (for example, co-registration) may be required prior to their use in a deep learning model [10]. Multi-modal fusion strategies can then be used to integrate the images to facilitate segmentation [11].

**Figure 1:**
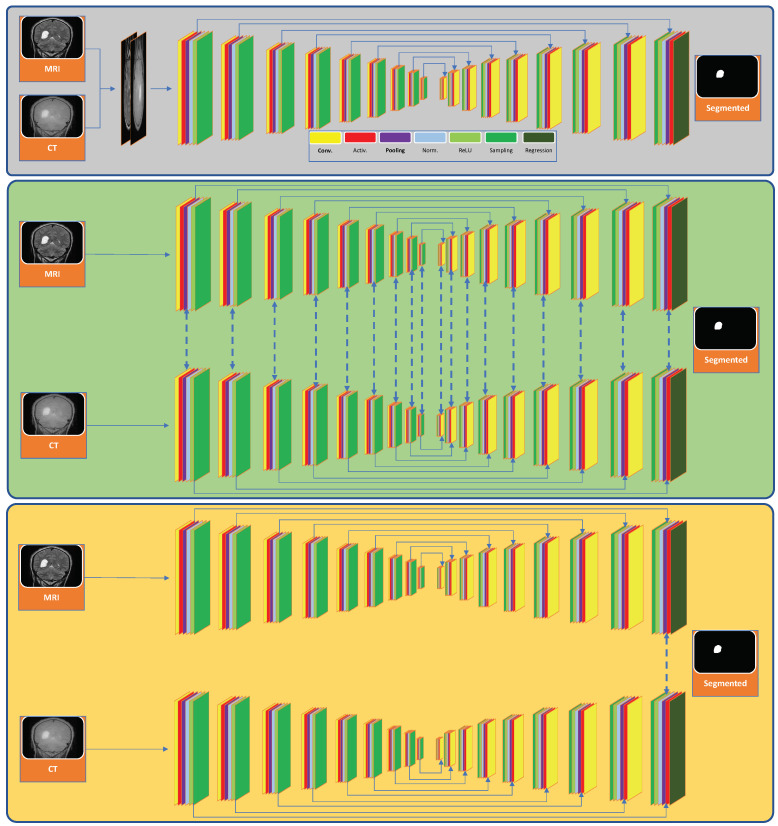
Multi-modal deep learning networks for the segmentation of brain tumors from computed tomography (CT) and magnetic resonance imaging (MRI) images. Here, the three sections (top to bottom) represent the input-, layer-, and decision-level fusion networks, respectively.

Fusion methods for deep-learning-based multi-modal image segmentation can broadly be categorized as: input-, layer-, and decision-level fusion [8]. In input-level fusion, multi-modal images are combined channel-by-channel and serve as multi-channel inputs, representing integrated image features [12]. For layer-level fusion, multi-modal images are used to train individual networks to perform segmentation. Those networks are interconnected at different layers to learn shared features from input images. In decision-level fusion, each image is given as input to its respective network, and the learned features are fused into a decision layer to perform the segmentation task. Figure 1 illustrates the differences between the three types of fusion methods.

Zhou et al. [13] used four different modalities of magnetic resonance imaging (MRI) images (T1-weighted, T2-weighted, contrast-enhanced (CE) T1-weighted, and fluid attenuation inversion recovery (FLAIR)) to perform multi-modal brain tumor segmentation using the BraTS [14] dataset. They used three convolutional neural networks (CNNs) in cascade to perform a coarse-to-fine segmentation in one pass. The initial network produced a coarse mask for the possible whole tumor region. The second network was used to predict the precise region of the whole tumor. The final network was used as post-processing to enhance the tumor region. They used three modified versions of the FusionNet [15] network architecture in the Caffe [16] platform. The three networks were identical except for their final segmentation layers. The authors used input-level fusion with an input patch size of 32-by-32-by-16-by-4, where the final dimension represented the four MRI modalities.

Similarly, Wang et al. [17] also proposed a multi-modal segmentation method using the BraTS dataset [14] to segment brain tumors. They identified the entire tumor, tumor core, and enhancing tumor core (the anatomical structure of the tumor) separately. To obtain a combined feature set, they combined the four modalities of MRI images (T1-weighted, T2-weighted, CE-T1-weighted, and FLAIR) as multi-channel inputs. Then, they divided their segmentation task into a sequence of three successive binary segmentation tasks according to the hierarchical framework of brain tumors. To avoid the complexity of a 3-D deep learning model, they trained three segmentation models for the three different orthogonal views (sagittal, axial, and coronal). They used the average of the predictions of the three models as the final prediction.

Guo et al. [9] proposed a method of lung tumor segmentation from MRI, computed tomography (CT), and positron emission tomography (PET). They performed multi-modal fusion strategies at the input, layer, and decision level. They concluded that performing image fusion at the input- and layer-levels was more beneficial than fusion at the decision-level. In order to develop and validate their method, they used a publicly available soft-tissue sarcoma (STS) dataset [18], which was initially generated from a well-known cancer imaging archive [19]. For input-level fusion, they considered 28-by-28-by-k image patches, where *k* represented the number of modalities in the input layer. In layer-level fusion, every modality’s network layers were learned from corresponding features independently. Then, they were used to train a single network to perform the segmentation task. For decision-level fusion, the output of each network’s prediction was used to decide the final segmentation through voting.

Choudhury et al. [20] investigated DeepLabv3+ [21] along with the Xception [22] CNN (as the base network) to perform brain tumor segmentation for multi-modal MRI images in the BraTS dataset. Similar to Wang et al. [17], they used various combinations of T1-weighted, T2-weighted, CE-T1-weighted, and FLAIR images to perform segmentation of the whole tumor, tumor core, and enhancing tumor. Instead of using the 3-D volume as an input to the network, they also used 2-D orthogonal slices extracted from it. They prepared the dataset as a three-channel input (using different combinations of orthogonal slices) for 18 DeepLabv3+ models. Then, they used the outputs of these models as inputs to three more DeepLabv3+ models to segment each of the three regions under consideration.

Sun et al. [6] investigated different kernel sizes in a 3-D fully convolutional neural network (FCNN) architecture for brain tumor segmentation. The input to their FCN was four-channel images formed from four different MRI modalities (T1-weighted, T2-weighted, CE-T1-weighted, and FLAIR). To reduce the sensitivity to network initialization and to speed up the training of the FCN, they introduced a 3-D batch normalization layer after every convolutional layer [23]. Their findings indicated that smaller kernel sizes may provide better segmentation performance.

In this paper, we explore different deep learning architectures and training/testing strategies for the multi-modal segmentation of brain tumors using a publicly available dataset. We enhance this dataset by developing a method of generating CT images from MRI. Using experimental results, we show that using generated CT images improves the performance of deep learning models.

## 2. Materials and Methods

**Figure 2:**
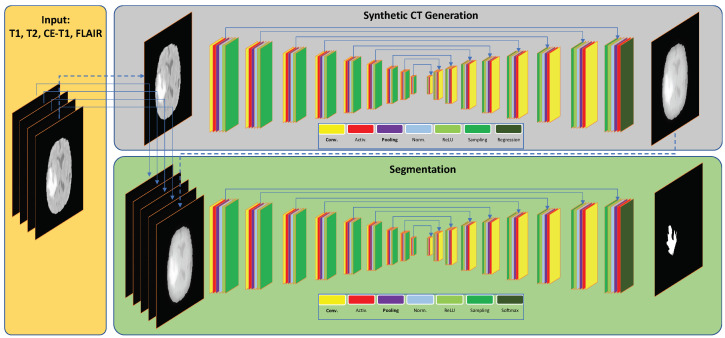
An overview of the proposed method. The first section (**top**) shows the process of synthetic CT generation from MRI. The second section (**bottom**) illustrates how the four MRI modalities along with the generated synthetic CT are used to perform the segmentation task.

Here, for the task of segmenting brain tumors using 3-D multi-modal images, we used data from a publicly available dataset that contained four types of MRI images. We investigated whether the segmentation accuracy could be improved by introducing an additional modality to this dataset, namely CT images. For this purpose, we developed a method of generating CT images from MRI. Figure 2 shows an overview of the proposed methodology.

### 2.1. Experimental Setup

The MATLAB numeric computing environment (academic), including the image processing and deep learning toolboxes, was used to implement all methods. An Intel Xeno Silver 4108 CPU (1.80 GigaHertZ) processor with 5 gigabytes of NVIDIA QuADro P2000 GPU graphics memory and 16 gigabytes of physical memory, running the education version of 64 bit Microsoft Windows, was used as the main experimental platform. Some experiments were conducted on the University of Melbourne’s high-performance computing (HPC) system [24].

### 2.2. Synthetic CT Generation

Generating one (synthetic) image modality from another is a convenient way to avoid intricacies involved in obtaining multiple images of the same patient. Pre-processing steps that enable the analysis of multiple images, such as co-registration, are also not required in this case. As such, there has been some interest in the field of synthetic data generation [25,26,27,28]. Here, we developed 2-D and 3-D CNNs for this purpose and used the retrospective image registration evaluation (RIRE) dataset (this dataset can be downloaded from: http://www.insight-journal.org/rire/download_data.php, accessed on 1 October 2021) containing the 3-D MRI and CT pairs of 16 patients to train them [29]. We used a modified U-Net [30,31] architecture, as it has been shown to perform well in similar tasks [27,32,33,34,35]. Prior to training, we co-registered each image pair using an existing multi-modal image registration method [36]. To train the 3-D CNN, we used the volumes themselves, while we used axial slices of the volumes to train the 2-D CNN.

As the existing dataset was relatively small, we augmented it by generating synthetic data based on the original data. To this end, we performed one of five different transformations (nil, rotate 90°, flip left–right, flip top–bottom, rotate 90°, and flip left–right) on each image, using the same transformation for each corresponding MRI and CT pair. To further augment the 2-D dataset of axial slices, we used random transformations (rotations in the range of [−20° 20°], translations in the range of [−1010], and reflections in the left–right and top–bottom directions). We used voxel patches of 32-by-32-by-13 (instead of the full volumes) in the 3-D CNN to avoid memory issues. We divided the dataset into three subgroups: training (70%), validation (15%), and testing (15%). Then, we optimized our training strategy with respect to this dataset.

**Figure 3:**
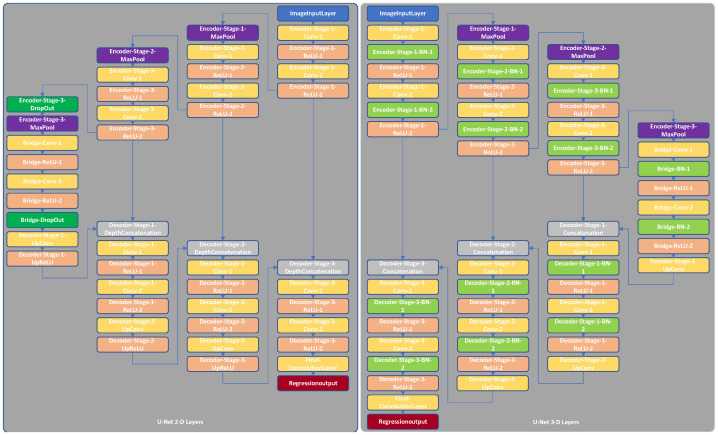
Two-dimensional (2-D) and three-dimensional (3-D) U-Net architectures with encoder depths of three.

A U-Net architecture typically consists of encoder and decoder sub-networks connected by a bridge. These sub-networks comprise multiple stages (depth) with each stage having multiple layers. There are two sets of convolutional (with additional batch normalization for 3-D) and rectified linear unit (ReLU) layers [37], followed by a 2-by-2 (or 2-by-2-by-2 for 3-D) max-pooling layer in each encoder sub-network stage. There is a transposed convolution layer for up-sampling, followed by two sets of convolutional and ReLU layers in each decoder sub-network stage. Another two sets of convolution (with additional batch normalization for 3-D) and ReLU layers are included in the bridge connection section. As the original U-Net architecture was developed for image segmentation, we replaced the softmax and segmentation output layers with a regression output layer to enable image-to-image regression. Examples of 2-D and 3-D U-Net architectures with depths of three are illustrated in Figure 3.

To optimize our network, we first tested depths from two to five and selected a depth of three, as it showed the best performance. Then, we used different training optimizers (stochastic gradient descent with momentum (SGDM) [38], root mean square propagation (RMSProp), and Adam [39]) with multiple training settings. We used root-mean-squared-error (RMSE) as the loss function along with different initial learning rates (0.1, 0.01, 0.001, and 0.0001). We chose 0.001 as the initial learning rate as it provided more balanced training and validation results after a few complete training cycles. To avoid memory issues, we set a minimum batch with one observation at each iteration. This minimum batch was used to evaluate the loss function gradient and update the training weights. The maximum number of epochs (complete passes over the training set) was set to 10 and was increased by a factor of 2 until it reached 160 and then increased to 200. We selected the weights from the network trained on the combination of loss function and maximum number of epochs that had the lowest validation loss.

### 2.3. Tumor Segmentation

Several state-of-the-art deep learning network architectures, mostly CNNs, dominate the field of medical image segmentation [40,41,42]. We considered two such CNNs: 2-D U-Net [30] and 3-D U-Net [31]. These were chosen as the best for our task after multiple training and testing strategies with different CNN architectures (AlexNet [43], VGG-16 and 19 [44], ResNet-18 and 50 [45], Inception-ResNet-v2 [46], DenseNet-201 [47], and DeepLabv3+ [21]).

**Figure 4:**
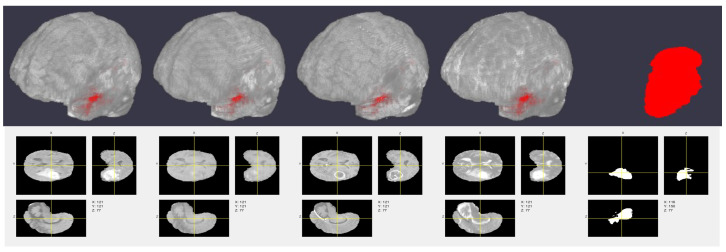
Example 3-D images and labels. From left to right: T1-weighted, T2-weighted, contrast-enhanced (CE)-T1-weighted, FLAIR, and labels. Corresponding orthogonal slices are shown in the second row.

We used the publicly available BraTS dataset (this dataset can be downloaded from: http://medicaldecathlon.com/, accessed on 1 October 2021) [48] in this experiment. It contains 750 MRI images (T1-weighted, T2-weighted, CE-T1-weighted, and FLAIR) of gliomas, the most commonly occurring brain malignancy. Figure 4 shows some images from this dataset along with their corresponding labels and orthogonal slices. From this dataset, we only used the 484 training volumes, as the remaining (test) data were not labeled. As in the case of CT generation (Section 2.2), we used the full volumes for the 3-D CNN and axial slices for the 2-D CNN. We augmented this dataset using the same strategies discussed in Section 2.2 (except for co-registration, as the dataset was already co-registered). Percentages of 70%, 15%, and 15% of the data were used for training, validation, and testing, respectively.

For the 3-D CNN, we used volume patches of 32-by-32-by-32 as input and output. For the 2-D CNN, we pre-processed the data to remove slices with a large number of background pixels (those with less than 120 labeled pixels). We used the remaining slices (25,051 out of a total of 60,729) in the training and testing of the 2-D U-Net.

First, we used the four different MRI scan modalities (T1-weighted, T2-weighted, CE-T1-weighted, and FLAIR) together to perform the segmentation task. Then, we paired each of the MRI modalities with the synthetic CT to obtain four combinations of input. Finally, we combined all five modalities to form a new set of inputs. We modified the input layer to allow for the respective number of channels (four, two, or five for the above input sets, respectively). The 2-D and 3-D U-Net architectures used for segmentation were the same as those shown in Figure 3 except that their regression output layers were replaced with softmax and segmentation output layers. The training strategies for segmentation were identical to those discussed in Section 2.2 except for the loss function. We used cross entropy as well as Tversky and generalized Dice loss functions [49,50]. The two latter loss functions were selected because they have proven effective for imbalanced datasets such as ours.

Additionally, we explored different fusion strategies: input-, layer-, and decision-level. For input-level fusion, we used one network with input images fused (see Figure 1 for an example). For layer- and decision-level fusion, we aligned two identical networks side by side and used a concatenation layer to connect them. For layer-level fusion, we connected the networks after their first ReLU layers to the second last ones iteratively. We chose to connect them after the second ReLU layers as it gave the most robust results. For decision-level fusion, we connected the networks right after the last ReLU layers. The following layers (of the combined network) after the connections were left unchanged.

## 3. Results

### 3.1. Synthetic CT Generation

**Figure 5:**
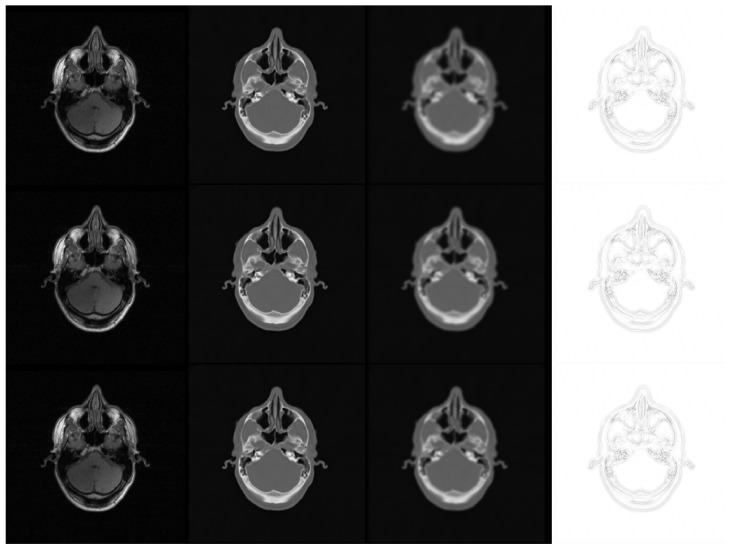
Example results for synthetic CT generation. The first, second, and third rows represent SGDM, RMSProp, and Adam optimizers, respectively. Columns from left to right show the input MRI, corresponding ground truth CT, predicted CT, and the complement of the absolute difference image for each optimizer.

Metrics such as the structural similarity (SSIM) index, peak signal-to-noise ratio (PSNR), and mean-squared error (MSE) [51] provide quantitative measures for the similarity between two images. We used these to compare the synthetic CT images generated by our method to ground truth CTs. Table 1 shows the performance (mean and standard deviation of a five-fold cross validation) of the 2-D and 3-D models with respect to different training optimizers. Note that the higher the value of SSIM and PSNR, the better the performance, while the opposite is true for MSE. Figure 5 shows some example outputs for the different training optimizers.

### 3.2. Tumor Segmentation

#### 3.2.1. Evaluation Metrics

The confusion matrix compares segmentation results with ground truth data and presents true positives (TP), true negatives (TN), false positives (FP), and false negatives (FN) in a C-by-C matrix (for C segmentation classes). We used several variants of some segmentation performance metrics based on the confusion matrix (Equations (1) to (3)) in our study: global accuracy, mean accuracy, mean intersection over union (IoU), weighted IoU, and mean BF (boundary F1) (additional information on these metrics can be obtained from: https://au.mathworks.com/help/vision/ref/evaluatesemanticsegmentation.html, accessed on 1 October 2021).

(1) $$\begin{array}{ccc} Accuracy & = & \frac{TP+TN}{TP+FN+FP+TN} \end{array}$$

(2) $$\begin{array}{ccc} IoUscore & = & \frac{TP}{TP+FP+FN} \end{array}$$

(3) $$\begin{array}{ccc} BF(boundaryF1) & = & 2\times \left(\frac{\frac{TP}{TP+FP}\times \frac{TP}{TP+FN}}{\frac{TP}{TP+FP}+\frac{TP}{TP+FN}}\right) \end{array}$$

Global accuracy, the accuracy over all classes, and mean accuracy, the average over all classes, provide computationally inexpensive and quick estimations. IoU (or Jaccard similarity coefficient) is one of the most commonly used metrics, with the mean IoU representing the average IoU over all classes. Weighted average IoU (weighted by the number of pixels per class) is useful when class sizes are imbalanced. The BF score has been seen to correlate better with human perception, as the contour of the BF matching score shows how well the predicted boundary of each class aligns with the actual boundary [52]. A mean BF score is the average BF score of all classes in all images of a dataset.

#### 3.2.2. Comparison of Different Loss Functions

**Figure 6:**
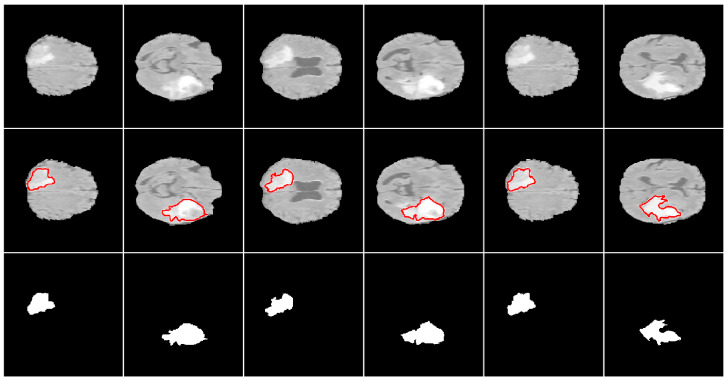
Example 2-D segmentation results. From top to bottom: input images, segmentation boundaries, and segmented regions.

We observed that the Adam optimizer performed best in the tumor segmentation task. As such, we tested its performance on different loss functions with all four MRI modalities of the original dataset provided as different input channels. Table 2 shows the results (mean and standard deviation of a five-fold cross validation). Figure 6 shows some 2-D segmentation results along with their segmentation boundaries and segmented regions. Similar to previous studies where the segmented region was small with respect to the background [53], the Dice loss function performed best. The 3-D methods showed more precise results than their 2-D counterparts, likely because volumetric data retain 3-D information that is lost when using 2-D slices separately as input.

#### 3.2.3. Comparison of Different Fusion Strategies

Using the network that showed the best results from the previous section (3-D U-Net with Adam optimizer and Dice loss function), we compared the effect of different fusion strategies on tumor segmentation performance. To this end, we considered all five image modalities (original MRI images as well as the synthetic CT we generated) together. As seen in Table 3, input-level fusion showed the best results when compared to layer- and decision-level fusion. This has been observed in other studies and could be due to the fact that earlier fusion may enable the network to learn shared representations, enabling it to learn better correlations across modalities [54].

#### 3.2.4. Comparison with Synthetic CT

**Figure 7:**
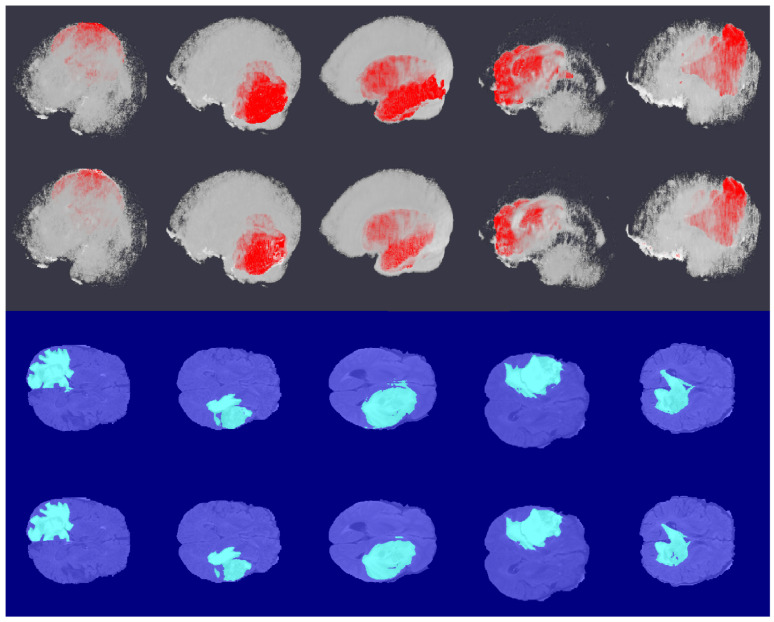
Example 3-D segmentation results. From top to bottom: ground truth labeled volume, predicted labeled volume, center slice of the ground truth labels, and center slice of the predicted labels. For the first and second columns, the brain volume was made transparent make the tumor region visible. The third and fourth columns represent the center slice of the ground truth and predicted labels along the depth direction.

Here, we evaluated the segmentation performance for the different MRI and synthetic CT combinations for input-level fusion, as it performed best in the previous experiment. Table 4 shows the results of this experiment, and Figure 7 visualizes some 3-D segmentation results. We observed that the combination of all five modalities performed best. This implies that our CT generation method was successful in providing additional information to supplement the segmentation task.

#### 3.2.5. Comparison of Different Network Architectures

We modified several state-of-the-art deep neural network architectures (AlexNet [43], VGG-16 and 19 [44], ResNet-18 and 50 [45], Inception-ResNet-v2 [46], DenseNet-201 [47], and DeepLabv3+ [21]) to accept 3-D images and perform the tumor segmentation task. For DeepLabv3+ [21], we used ResNet-18 [45], ResNet-50 [45], and Inception-ResNet-v2 [46] as base networks. As DeepLabv3+ [21] with ResNet-50 [45] showed the best results, we used that in our experiment. As seen from Table 5, the proposed method outperformed all of the other considered networks.

#### 3.2.6. Comparison with Similar Methods

We compared the proposed method with similar existing methods (discussed earlier). We re-implemented these methods and in some cases, modified their architectures to suit our dataset. For example, for the network presented by Guo et al. [9], we used input-level fusion as they observed it generally worked better. For the network introduced by Sun et al. [6], we used a kernel size of 5-by-5-by-5 as they found it showed higher segmentation performance. In all other cases, we used their baseline methods. As seen from Table 6, the proposed method performed better than the others on every metric. This could be due to the fact that we introduced new information in the form of a new image modality to the dataset and because we optimized our method systematically.

## 4. Conclusions

We discussed how a segmentation task, namely brain tumor segmentation, can be improved by (1) adding a synthetic CT modality to an existing MRI dataset and (2) optimizing network configurations. To generate synthetic CT images, we used a modified U-Net architecture. We experimentally demonstrated that the proposed method outperformed other similar state-of-the-art methods. In future work, we will explore how this work can be extended to other segmentation tasks/datasets. We will also investigate how other complex CNN architectures can be utilized for this purpose. Furthermore, we will consider multi-class segmentation, for example, to identify different regions of a tumor. A natural extension to this would be to classify segmented areas (for example, to identify the type of tumor). We also aim to explore how our synthetic image generator could be utilized for other modalities (for example, to obtain T1-weighted MRI images from T2-weighted ones).

## Figures and Tables

**Table 1:** Synthetic computed tomography (CT) generation performance (mean and standard deviation of a five-fold cross validation). Best results for each metric are shown in bold.

Dimension	Optimizer	SSIM	PSNR	MSE
2-D	SGDM	0.9163 ± 0.021	29.0613 ± 0.668	80.7135 ± 1.856
	RMSProp	0.9459 ± 0.018	31.0253 ± 0.621	51.3513 ± 1.027
	Adam	0.9617 ± 0.009	32.5197 ± 0.309	36.4010 ± 0.346
3-D	SGDM	0.9588 ± 0.023	32.5921 ± 0.766	35.7988 ± 0.841
	RMSProp	0.9746 ± 0.018	35.0689 ± 0.663	20.2392 ± 0.383
	Adam	**0.9827 ± 0.009**	**36.9242 ± 0.351**	**13.2027 ± 0.125**

**Table 2:** Segmentation performance (mean and standard deviation of a five-fold cross validation) with different loss functions. Best results for each metric are shown in bold.

Dimension	Loss Function	Global Accuracy	Mean Accuracy	Mean IoU	Weighted IoU	Mean BF
2-D	Cross Entropy	0.9525 ± 0.028	0.9464 ± 0.027	0.7312 ± 0.024	0.9578 ± 0.028	0.7512 ± 0.023
	Tversky Loss	0.9530 ± 0.028	0.9465 ± 0.027	0.7415 ± 0.023	0.9580 ± 0.028	0.7655 ± 0.023
	Dice Loss	0.9542 ± 0.027	0.9473 ± 0.026	0.7450 ± 0.024	0.9574 ± 0.027	0.7683 ± 0.024
3-D	Cross Entropy	0.9549 ± 0.025	0.9378 ± 0.022	0.7409 ± 0.023	0.9606 ± 0.026	0.7987 ± 0.023
	Tversky Loss	0.9545 ± 0.024	0.9485 ± 0.021	0.7875 ± 0.022	0.9613 ± 0.026	0.8035 ± 0.021
	Dice Loss	**0.9580 ± 0.024**	**0.9520 ± 0.020**	**0.8170 ± 0.021**	**0.9625 ± 0.025**	**0.8085 ± 0.020**

**Table 3:** Segmentation performance (mean and standard deviation of a five-fold cross validation) with different fusion strategies. Best results for each metric are shown in bold.

Fusion Level	Global Accuracy	Mean Accuracy	Mean IoU	Weighted IoU	Mean BF
Decision	0.8842 ± 0.029	0.8690 ± 0.028	0.6828 ± 0.022	0.8725 ± 0.028	0.7074 ± 0.023
Layer	0.9053 ± 0.020	0.8832 ± 0.020	0.6957 ± 0.016	0.8968 ± 0.021	0.7290 ± 0.017
Input	**0.9849 ± 0.009**	**0.9579 ± 0.008**	**0.8410 ± 0.009**	**0.9706 ± 0.010**	**0.8986 ± 0.009**

**Table 4:** Segmentation performance (mean and standard deviation of a five-fold cross validation) for different combinations of magnetic resonance imaging (MRI) and synthetic CT. Best results for each metric are shown in bold.

CT With	Global Accuracy	Mean Accuracy	Mean IoU	Weighted IoU	Mean BF
T1-weighted	0.9405 ± 0.012	0.9255 ± 0.012	0.7138 ± 0.009	0.9140 ± 0.011	0.7450 ± 0.009
T2-weighted	0.9415 ± 0.012	0.9218 ± 0.012	0.7174 ± 0.009	0.9145 ± 0.011	0.7455 ± 0.009
CE-T1-weighted	0.9418 ± 0.012	0.9263 ± 0.012	0.7181 ± 0.009	0.9157 ± 0.011	0.7468 ± 0.009
FLAIR	0.9422 ± 0.012	0.9281 ± 0.012	0.7153 ± 0.009	0.9184 ± 0.011	0.7480 ± 0.009
All	**0.9849 ± 0.009**	**0.9579 ± 0.008**	**0.8410 ± 0.009**	**0.9706 ± 0.010**	**0.8986 ± 0.009**

**Table 5:** Segmentation performance (mean and standard deviation of a five-fold cross validation) with different network architectures. Best results for each metric are shown in bold.

Network Architecture	Global Accuracy	Mean Accuracy	Mean IoU	Weighted IoU	Mean BF
AlexNet [43]	0.8847 ± 0.027	0.8408 ± 0.025	0.7505 ± 0.023	0.8595 ± 0.026	0.7682 ± 0.023
VGG-16 [44]	0.9238 ± 0.026	0.8857 ± 0.026	0.7750 ± 0.022	0.9074 ± 0.026	0.8185 ± 0.024
VGG-19 [44]	0.9355 ± 0.028	0.8920 ± 0.027	0.7852 ± 0.024	0.9217 ± 0.028	0.8250 ± 0.025
ResNet-18 [45]	0.9641 ± 0.020	0.9287 ± 0.019	0.8074 ± 0.017	0.9430 ± 0.020	0.8583 ± 0.018
ResNet-50 [45]	0.9715 ± 0.019	0.9404 ± 0.019	0.8170 ± 0.016	0.9543 ± 0.019	0.8732 ± 0.017
Inception-ResNet-v2 [46]	0.9735 ± 0.019	0.9460 ± 0.018	0.8175 ± 0.016	0.9542 ± 0.019	0.8842 ± 0.018
DeepLabv3+ [21]	0.9825 ± 0.009	0.9505 ± 0.009	0.8358 ± 0.008	0.9680 ± 0.009	0.8963 ± 0.009
DenseNet-201 [47]	0.9782 ± 0.015	0.9490 ± 0.014	0.8325 ± 0.012	0.9590 ± 0.014	0.8783 ± 0.013
Proposed	**0.9849 ± 0.009**	**0.9579 ± 0.008**	**0.8410 ± 0.009**	**0.9706 ± 0.010**	**0.8986 ± 0.009**

**Table 6:** Segmentation performance (mean and standard deviation of a five-fold cross validation) with similar methods. Best results for each metric are shown in bold.

Network Architecture	Global Accuracy	Mean Accuracy	Mean IoU	Weighted IoU	Mean BF
Zhou et al. [13]	0.9753 ± 0.008	0.9557 ± 0.008	0.8305 ± 0.007	0.9684 ± 0.008	0.8855 ± 0.008
Wang et al. [17]	0.9825 ± 0.008	0.9560 ± 0.007	0.8384 ± 0.006	0.9688 ± 0.007	0.8873 ± 0.007
Guo et al. [9]	0.9814 ± 0.008	0.9531 ± 0.007	0.8305 ± 0.006	0.9654 ± 0.008	0.8824 ± 0.007
Choudhury et al. [20]	0.9810 ± 0.008	0.9515 ± 0.007	0.8375 ± 0.006	0.9650 ± 0.007	0.8955 ± 0.007
Sun et al. [6]	0.9780 ± 0.010	0.9435 ± 0.009	0.8190 ± 0.008	0.9650 ± 0.009	0.8385 ± 0.008
Proposed	**0.9849 ± 0.009**	**0.9579 ± 0.008**	**0.8410 ± 0.009**	**0.9706 ± 0.010**	**0.8986 ± 0.009**

## Data Availability

The datasets used in this study are freely available. Associated code can be accessed from https://github.com/khtohidulislam/ImageSegmentationCodes, accessed on 4 January 2022.

## Abbreviations

The following abbreviations are used in this manuscript:

2-D	two-dimensional
3-D	three-dimensional
BF	boundary F1
CE	contrast-enhanced
CNN	convolutional neural network
CT	computed tomography
FCNN	fully convolutional neural network
FLAIR	fluid attenuation inversion recovery
FN	false negatives
FP	false positives
HPC	high-performance computing
IoU	intersection over union
MRI	magnetic resonance imaging
MSE	mean-squared error
PET	positron emission tomography
PSNR	peak signal-to-noise ratio
ReLU	rectified linear unit
RIRE	retrospective image registration evaluation
RMSE	root-mean-squared-error
RMSProp	root mean square propagation
SGDM	stochastic gradient descent with momentum
SSIM	structural similarity
STS	soft-tissue sarcoma
TN	true negatives
TP	true positives
